# Rethinking the role of alpha toxin in *Clostridium perfringens*-associated enteric diseases: a review on bovine necro-haemorrhagic enteritis

**DOI:** 10.1186/s13567-017-0413-x

**Published:** 2017-02-16

**Authors:** Evy Goossens, Bonnie R. Valgaeren, Bart Pardon, Freddy Haesebrouck, Richard Ducatelle, Piet R. Deprez, Filip Van Immerseel

**Affiliations:** 1 Department of Pathology, Bacteriology and Avian Diseases, Faculty of Veterinary Medicine, Ghent University, Salisburylaan 133, B-9820 Merelbeke, Belgium; 2 Department of Large Animal Internal Medicine, Faculty of Veterinary Medicine, Ghent University, Salisburylaan 133, B-9820 Merelbeke, Belgium

## Abstract

Bovine necro-haemorrhagic enteritis is an economically important disease caused by *Clostridium perfringens* type A strains. The disease mainly affects calves under intensive rearing conditions and is characterized by sudden death associated with small intestinal haemorrhage, necrosis and mucosal neutrophil infiltration. The common assumption that, when causing intestinal disease, *C. perfringens* relies upon specific, plasmid-encoded toxins, was recently challenged by the finding that alpha toxin, which is produced by all *C. perfringens* strains, is essential for necro-haemorrhagic enteritis. In addition to alpha toxin, other *C. perfringens* toxins and/or enzymes might contribute to the pathogenesis of necro-haemorrhagic enteritis. These additional virulence factors might contribute to breakdown of the protective mucus layer during initial stage of pathogenesis, after which alpha toxin, either or not in synergy with other toxins such as perfringolysin O, can act on the mucosal tissue. Furthermore, alpha toxin alone does not cause intestinal necrosis, indicating that other virulence factors might be needed to cause the extensive tissue necrosis observed in necro-haemorrhagic enteritis. This review summarizes recent research that has increased our understanding of the pathogenesis of bovine necro-haemorrhagic enteritis and provides information that is indispensable for the development of novel control strategies, including vaccines.

## Introduction

Bovine necro-haemorrhagic enteritis caused by *Clostridium perfringens* is an important cause of sudden death with necro-haemorrhagic lesions in the small intestine. The disease typically affects calves in good to excellent body condition that are fed large amounts of milk or milk replacer, often without premonitory signs of illness. Although morbidity is rather low, mortality is close to 100%, making it an economically important disease [1]. Despite the economic importance of the disease, until recently little was known about the pathogenesis of bovine necro-haemorrhagic enteritis. In the last few years, a series of papers have been published providing new insights in *C. perfringens*-associated bovine necro-haemorrhagic enteritis. Here, we review the knowledge on bovine necro-haemorrhagic enteritis, present a hypothetical model on the pathogenesis and discuss the current problems in vaccination.

## Aetiology

*Clostridium perfringens* ranks amongst the most widespread bacteria, with an ubiquitous environmental distribution in soil, sewage, food, faeces, and the normal intestinal microbiota of humans and animals. This Gram-positive, anaerobic spore former is, however, also one of the most common pathogens, causing a spectrum of important human and animal diseases, ranging from myonecrotic to enteric infections [2, 3]. The virulence of *C. perfringens* is mediated by its intimidating arsenal of toxins and degradative enzymes. As a species, *C. perfringens* produces at least 16 toxins and extracellular enzymes [3–5]. However, no single strain produces this entire toxin panoply, resulting in considerable variation in the repertoire of toxins and degradative enzymes produced by different strains of this bacterium. These strain-to-strain differences in toxin production permits the classification of *C. perfringens* isolates into five toxinotypes (A, B, C, D and E), based on the presence of genes encoding four so-called major toxins: alpha, beta, epsilon and iota toxin [3]. Besides expressing one or more of the typing toxins, *C. perfringens* strains can produce additional toxins, including, but not limited to, enterotoxin and necrotic enteritis B-like toxin (NetB), which are also very important during certain diseases, for example human food poisoning or necrotic enteritis in broiler chickens [5, 6].

*C. perfringens* type A strains are the suspected aetiological agent of multiple bovine alimentary tract disorders. From these diseases, clostridial abomasitis and necro-haemorrhagic enteritis show remarkable similarities in aetiology, clinical symptoms, histological findings and predisposing factors. Even now, it is not clear whether they are truly different diseases or whether they should be considered as clinical or pathological variants of the same disease. For the completeness of this review, both diseases are included.

There is recent evidence clearly demonstrating that bovine necro-haemorrhagic enteritis is caused by *C. perfringens* type A strains. Indeed, the intestinal disease was reproduced by inoculation of bovine intestinal ligated loops with type A strains isolated from necro-haemorrhagic enteritis cases [7–9]. Furthermore, the causative role of *C. perfringens* type A in clostridial abomasitis was confirmed when intraruminal administration of *C. perfringens* type A to neonatal calves induced clinical signs similar to naturally acquired disease [10]. Almost exclusively toxinotype A strains are isolated from animals diagnosed with either necro-haemorrhagic enteritis [11–14] or clostridial abomasitis [10, 15, 16]. However, the involvement of this toxinotype and its toxin(s) was and still is heavily debated. As type A strains can be present in the normal intestinal microbiota, isolation of this toxinotype is not diagnostic for disease. Also detection of its major toxin, alpha toxin, has little diagnostic value, as it can be present in the faeces of healthy animals [17]. Therefore, diagnosis of enteric type A disease is not straightforward. Furthermore, also other toxinotypes can cause disease in cattle. *C. perfringens* type C can cause sudden death in neonatal calves less than 10 days of age [18]. The intestinal lesions are similar to those described for type A, with severe necrosis and haemorrhages in the small intestine and neutrophil infiltration [19, 20]. *C. perfringens* type B and E only sporadically cause disease. Only one report describing *C. perfringens* type B-associated disease in cattle was found. This report provides only limited information, but bloody diarrhoea, haemorrhagic enteritis and haemorrhages in all vital organs were described [21]. *C. perfringens* type E is considered an infrequent cause of haemorrhagic enteritis and sudden death in neonatal calves [22], however, one report also describes type E enterotoxaemia in adult cows [23]. Much research is focussed on the pathology caused by *C. perfringens* type D strains. Although *C. perfringens* type A and type D strains cause completely different pathologies, they both are commonly described as enterotoxaemia, making the nomenclature confusing [7, 9, 12, 21, 22, 24]. The term “enterotoxaemia” is widely applied to various diseases caused by *C. perfringens*, but it is appropriate only for diseases in which the major signs are caused by systemic actions of the toxins [25]. Indeed, type D enterotoxaemia is characterised by neurological signs without the presence of major intestinal lesions [26]. This is in contrast to the disease caused by type A strains, which is characterised by intestinal necrosis and haemorrhages, with neurological effects only sporadically being reported [27]. Therefore, we prefer to describe this syndrome as bovine necro-haemorrhagic enteritis, thereby making a clear distinction between the pathologies caused by *C. perfringens* type A or D, and clearly describing the lesions caused by the pathogen.

## Epidemiology

### Prevalence

Clostridial infection of the gastro-intestinal tract of cattle is a common problem all around the world [15, 16, 28–30]. *C. perfringens* type A disease usually presents as individual sporadic cases. In young calves, *C. perfringens* type A disease mostly is characterized by abomasitis, often with lesions also in the upper part of the small intestine [16, 31]. In older calves, *C. perfringens* type A causes necro-haemorrhagic enteritis. This disease can occur in cattle of all ages, but mainly affects suckling and veal calves in good to excellent body condition of up to four [1, 12, 29] and eight months of age [9, 32] respectively. Although the disease is apparently widespread and the overall prevalence is unknown, there are differences in incidence between different breeds and production systems. Accurate data on the prevalence are only available for the disease in Belgium, where a mortality rate of 4.7% (accounting for approximately 50% of total mortality) has been reported in suckling calves [29]. The majority of the affected animals (89%) are of the double muscled Belgian Blue beef cattle breed, suggesting a possible genetic influence for the susceptibility to necro-haemorrhagic enteritis. However, dairy breed calves are separated from the cow as soon as possible after birth and are typically not raised as suckling calves. Hence, as the majority of the affected animals are suckling calves, dietary influences can also be responsible for the difference in disease susceptibility. Also in veal calves, predominantly beef cattle breeds are affected, accounting for 20% of total mortalities on average, compared to 4% in dairy and mixed breed veal calves [1, 32]. In addition to a possible breed influence, dietary differences between veal production systems are suspected to have a great effect as an eliciting factor [1, 32]. Whereas dairy breed veal calves receive milk powders with very little animal protein, beef cattle breeds receive a high amount of skimmed milk powder. An important risk period for bovine enterotoxaemia is situated at the end of the production cycle, where calves are fed high amounts of highly concentrated milk proteins [32]. Whereas dairy or traditional beef calves receive on average a maximum of 6 litres milk replacer per day at a concentration of 125 g/L, beef cattle breed veal calves receive at the end of fattening as much as 16 L daily, at a concentration ranging from 150 to 190 g/L. The predisposition of these calves may thus be linked to their higher feed, protein and energy intake.

### Predisposing factors

#### Nutrition

Since *C. perfringens* lacks many genes necessary for amino acid biosynthesis, it cannot grow in an environment where a specific amino acid supply is limited [33]. High dietary levels of digestible carbohydrates that exceed the digestion and absorption capacity of the intestinal mucosa can be utilized by *C. perfringens* to proliferate [34]. Indeed, previous studies have suggested that protein- and energy-rich diets predispose for this disease [1]. Dietary issues such as changes in feed composition, feed quantity, bringing animals to pasture or moving to a different pasture are often noted 24–36 h prior to death due to necro-haemorrhagic enteritis [29]. Also the ad libitum provision of concentrate feed to suckling calves predisposes for necro-haemorrhagic enteritis. The predisposition of both spring grass and feed concentrates might be attributed to the high protein concentration and low amount of fibres, which may alter the microbiota composition and favour clostridial overgrowth. By contrast, high fibre diets are often believed to protect from gastro-intestinal disease. Indeed, the reduction of the amount of concentrates provided to suckling calves and addition of dietary fibres reduced the incidence of necro-haemorrhagic enteritis [29]. In addition to feed concentrates and pasture, also cow’s milk or milk replacer in the diet seems to predispose for *C. perfringens*-associated enteric diseases. Indeed, necro-haemorrhagic enteritis is more frequently observed in veal calves and suckler calves, and also neonatal calves are prone to abomasitis [1, 16, 27, 32]. A common dietary factor in these populations is the high proportion of cow’s milk or milk replacer in the diet. The whey present in cow’s milk or milk replacer contains high quality, readily available amino-acids, potentially predisposing for clostridial overgrowth. We showed that milk replacer has an important predisposing effect in a ligated loop model for necro-haemorrhagic enteritis [35]. Furthermore, within the Belgian Blue breed, milk-fed veal calves produce less antibodies against *C. perfringens* alpha toxin than beef calves [36]. This suggests that calves fed cow’s milk or milk replacer might have less contact with alpha toxin, leading to the absence of active immunity, and thus potentially leaving the calves unprotected against necro-haemorrhagic enteritis. Indeed, when *C. perfringens* is cultured in the presence of milk replacer, the alpha toxin activity of the supernatant decreases in a dose-dependent manner, compared to a negative control cultured without milk replacer [36, Supplemental file 1]. The observation that contact with milk decreases toxin expression seems in contradiction to the observation that high milk diets predispose for necro-haemorrhagic enteritis. However, the effect of milk on the *C. perfringens* production of mucinases, sialidases or other colonizing factors has not been investigated and might be of importance. Furthermore, the presence of crude proteins in the diet increases the mucin concentration in the small intestine [37]. This might favour colonization of the small intestinal mucus layer by *C. perfringens* and subsequent localized toxin production. Up till now the molecular mechanism behind the onset of necro-haemorrhagic enteritis is unknown. Furthermore, the role of feed in the pathogenesis of necro-haemorrhagic enteritis cannot be attributed to one specific factor, but to a very complex interaction of influences from a broad variety of fed components and their effect on and interaction with bacterial factors.

#### Stress and intestinal homeostasis

Stressful environmental conditions, such as regrouping, transport, handling and medical treatments have been mentioned as risk factors for necro-haemorrhagic enteritis [1]. A post-stress modification of the intestinal microbiota by induction of a paralytic ileus is a well-known phenomenon [38–40]. This can lead to unadapted digestive processes, and consequently a higher availability of these nutrients for bacterial growth [41]. The finding of Manteca et al. that ganglia are degenerated in necro-haemorrhagic enteritis suggests a direct effect on intestinal motility, additional to the general paralytic effect of enteritis, leading to a vicious circle. The consequent intestinal stasis diminishes the flushing of bacteria and toxins, and can contribute to bacterial overgrowth and colonization of the intestinal mucus layer [29, 42].

## Clinical signs and lesions

### Clinical signs

One of the hallmarks of bovine necro-haemorrhagic enteritis and clostridial abomasitis is the speed of disease progression. In most cases, apparently healthy animals with an excellent body conformation are found dead. When premonitory signs prior to death are noticed by the farmer, death due to necro-haemorrhagic enteritis occurs within 5 h [9, 29]. In those cases, signs of cardiovascular shock, including a lateral recumbent position and cold extremities, are commonly observed. Less frequently, colic and respiratory distress are present, whereas nervous symptoms, distended abdomen and diarrhoea are rather rare [1, 9, 29]. Premonitory disease signs for clostridial abomasitis are less extensively described, but include colic and haemorrhagic diarrhoea [16, 43, 44].

### Macroscopic lesions

**Figure 1:**
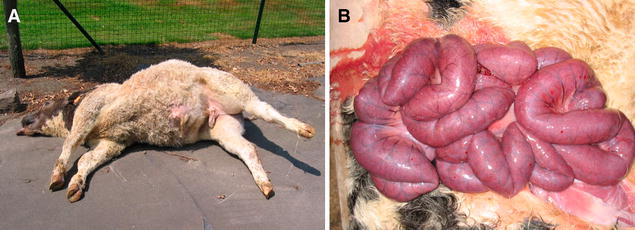
**Post-mortem, macroscopic presentation of a case of bovine necro-haemorrhagic enteritis. A** Acute death in a Belgian Blue calf with a distended abdomen and marked meteorism. **B** Severely dilated and congested small intestine of a case of bovine necro-haemorrhagic enteritis. When opening it is filled with blood.

Post-mortem a remarkable and rapid meteorism (bloating, tympanites…) of the abdomen and rapid putrefaction with foul smell is typical. On necropsy, cases of necro-haemorrhagic enteritis are characterized by diffuse or localized small intestinal haemorrhage with abundant liquid haemorrhagic contents (Figure 1) [1, 9, 29]. The haemorrhagic nature of the intestinal content is limited to the diseased intestinal segment, suggesting a paralytic ileus. The affected region can range from 10 cm to the entire length of the small intestine and is most often located in the jejunum [9, 29]. However, also the ileum and less frequently the caecum, colon or abomasum can be affected [29]. There is also gas accumulation and in the majority of the cases the mesenteric lymph nodes are enlarged. Less frequently, lesions in other internal organs, such as petechiae or congestion, which are typical for shock, are observed [1, 29]. In young calves lesions are mainly situated in the abomasum, whereby this syndrome is referred to as clostridial abomasitis. The lesions mainly comprise acute emphysematous and necrotizing haemorrhagic inflammation of the abomasal mucosa, with marked oedema in the lamina propria and submucosa. Similar lesions are frequently present in the rumen, reticulum and duodenum [10, 16, 43, 44].

### Microscopic lesions

**Figure 2:**
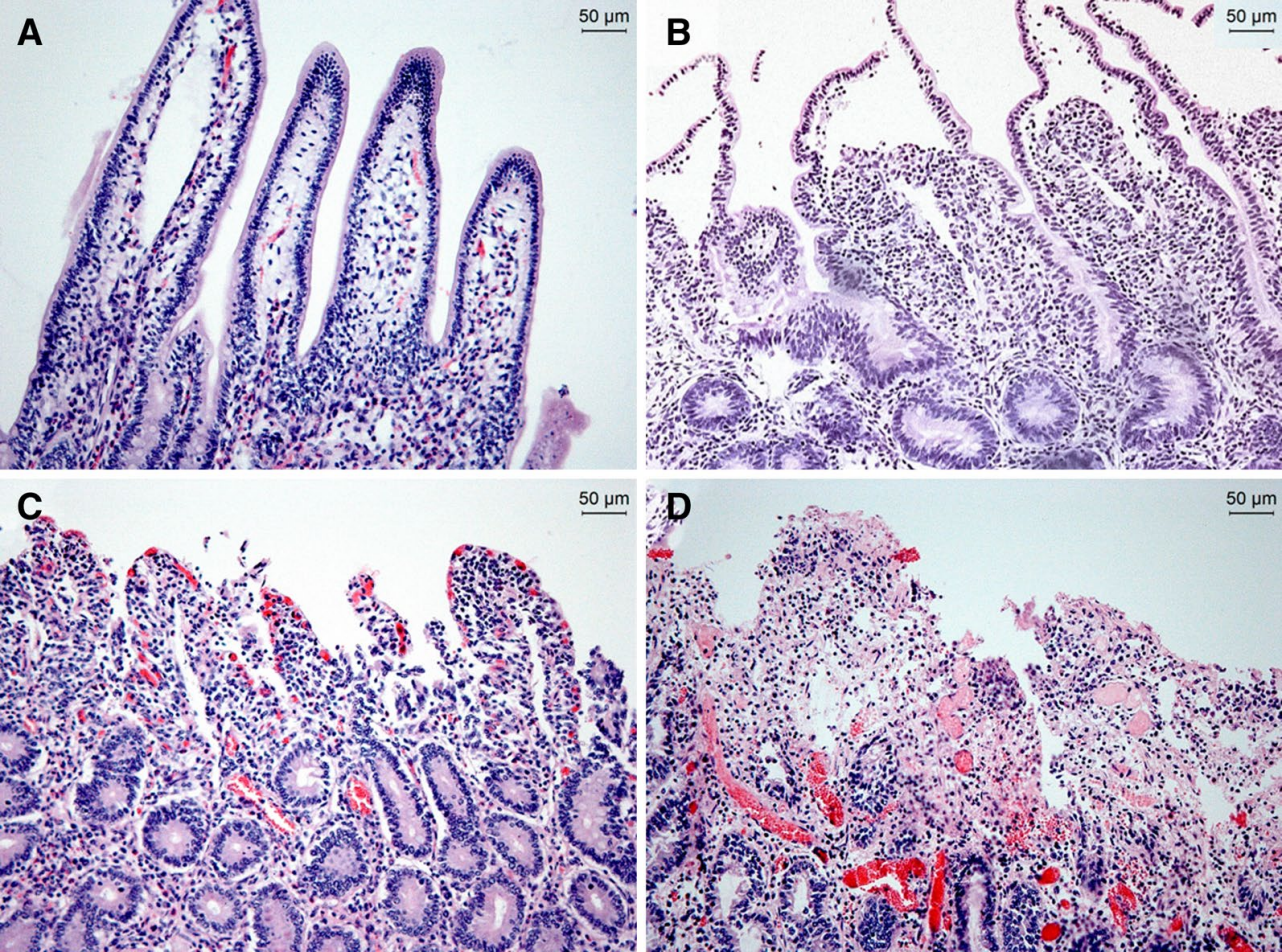
**Histological damage in the bovine small intestine treated with a** ***C. perfringens*** **necro-haemorrhagic enteritis isolate over time.** Ligated small intestinal loops were inoculated with sterile bacterial culture medium (**A**) or BCP62, a *C. perfringens* type A strain isolated from a case of bovine necro-haemorrhagic enteritis (**B**–**D**) [28]. Intestinal loops were injected with 30 min intervals, resulting in loops with different incubation times at time of sampling. Loops incubated with sterile bacterial culture medium for 5 h showed normal intestinal villi with a well-preserved epithelium and lamina propria (**A**). Histological damage, consisting of epithelial sloughing, is observed within 30 min after injection of *C. perfringens* (**B**). Complete loss of the epithelium and congestion of the capillaries was noted after 3 h incubation with *C. perfringens* (**C**). After 5 h, more severe haemorrhages and necrosis of the tips of the villi are observed (**D**).

Microscopic examination of necro-haemorrhagic enteritis cases reveals intestinal haemorrhages and cell necrosis extending from the tip of the villi to the base of the crypts, as well as infiltration of neutrophils and lymphocytes [1, 38]. In the intestinal lumen, clusters of *C. perfringens* bacteria can be found, localized in the necrotic areas. However, they are typically not found in the mucosa of the intestinal wall [12]. Lesions typical for toxaemia are not consistently present in internal organs [12, 38]. We recently gained more insight into the sequence of histopathological events during lesion development using a calf intestinal loop model of necro-haemorrhagic enteritis (Figure 2) [35]. In early stages, only congestion of the capillaries and epithelial sloughing are observed, with strings of viable epithelium present in the lumen. One can argue that the observed detachment of epithelial cells is a result of post-mortem autolysis. However, control samples showed no sloughed epithelium, indicating the pathological nature of this event, which is in accordance with early lesions observed in *C. perfringens*-induced necrotic enteritis in broilers [45]. When the disease progresses, oedema of the mucosa, infiltration of inflammatory cells, villus blunting and haemorrhages occur, with necrosis only observed in advanced stages of disease. When severe necrosis is present, the necrotic tips of the villi are clearly separated from the underlying viable tissue. These observations indicate that the villi initially are damaged at the basement membrane and lateral domain of the enterocytes, followed by extensive damage to the lamina propria.

In contrast to necro-haemorrhagic enteritis, descriptions of microscopic changes in clostridial abomasitis are scanty. For clostridial abomasitis, mucosal haemorrhages, necrosis and variable degrees of inflammatory cell infiltration in the abomasum, with marked oedema in the lamina propria and submucosa are generally reported [16, 43, 44]. Experimental reproduction of disease has confirmed these histopathologic observations [10].

## Virulence factors involved in disease

Although the role of other toxinotypes of *C. perfringens* in diseases originating in the intestine is well documented, the involvement of *C. perfringens* type A strains is rather controversial and often questioned. For many years, the involvement of *C. perfringens* type A strains in intestinal disorders was commonly accepted [3, 46–48]. More recently, the opinion in the scientific literature has shifted towards underrating the role of type A strains in enteric disease, leading to the postulation that type A strains are only important for myonecrotic infections (gas gangrene) and that enteric diseases in various animal species are generally caused by type B–E or type A subtypes, producing specific toxins such as enterotoxin and NetB [49, 50]. In analogy with the recent discovery of subtypes of type A strains that produce newly identified toxins which are (potentially) involved in intestinal diseases in pigs, poultry and horses, it was suspected that a disease- and/or species-specific toxin was essential for the pathogenesis of *C. perfringens* type A-associated enteric disease in cattle as well.

### Beta2 toxin, a prime suspect of enteric diseases?

Beta2 toxin is a pore-forming toxin that is associated with enteritis in neonatal pigs [51, 52] and gentamicin-associated diarrhoea in horses [53–55]. Beta2 toxin positive *C. perfringens* strains are widespread and can be isolated from various wild and domestic animals and humans, but also from food, soil and sludge [56–59]. *C. perfringens* type A strains harbouring the beta2 toxin gene have been isolated from diseased as well as healthy cattle of different ages [8, 59, 60]. However, there seem to be geographical differences in the reported number of beta2 toxin positive isolates and up to now no unequivocal correlation between the isolation of beta2 toxin positive strains and gastro-intestinal disease in either calves or adult cattle could be demonstrated [7, 27, 44, 59]. A possible role of beta2 toxin in bovine enterotoxaemia was suggested in 2002 when Manteca et al. induced necrotic lesions with a beta2 toxin producing type A strain in a ligated loop experiment [7]. However, this was only tested in one intestinal loop in one calf, and the strain also produced large amounts of alpha toxin. In addition, no isogenic beta2 toxin-deficient strain was used as a control. Therefore, effects of other factors cannot be excluded. More recently, Morris et al. and Valgaeren et al. were able to induce necrotic lesions in an intestinal loop model by inoculation of type A strains not producing the beta2 toxin [8, 35]. Taking these results together with the observation that there is no correlation between the isolation of beta2 toxin positive strains and the occurrence of disease [59], beta2 toxin seems not essential for the development of *C. perfringens* type A-associated gastro-intestinal diseases in cattle. However, a synergism between beta2 toxin and other toxins is plausible and should be further investigated [7, 60]. The actual role of beta2 toxin in other enteric diseases is also not clear and recent papers suggest a limited role of beta2 toxin in disease [61] and that beta2 toxin positive *C. perfringens* type A strains merely reflect the normal intestinal microbiota [62].

### Alpha toxin, a critical role in pathogenesis?

Alpha toxin is a phospholipase C enzyme, which is preferentially active towards phosphatidylcholine and sphingomyelin, two major components of the outer leaflet of eukaryotic cell membranes [63]. This toxin is produced by all *C. perfringens* strains, although type A strains usually produce higher amounts than other toxinotypes. Alpha toxin is the major toxin produced by type A strains, but its role in intestinal diseases is controversial and heavily debated. Indeed, for over 30 years it was believed that alpha toxin was the key virulence factor in necrotic enteritis caused by *C.* *perfringens* in broiler chickens, until it was shown that a novel toxin, NetB, was crucial for disease [5, 64]. This is in contrast to the situation in cattle where incubation of numerous strains from different origin and toxinotypes in bovine ligated intestinal loops induced similar necro-haemorrhagic lesions, suggesting that common virulence factors rather than disease-specific toxins are essential [35]. Furthermore, analysis of the complete genome sequence of a bovine clostridial abomasitis isolate failed to reveal novel toxin genes [65]. Therefore a possible role of commonly produced toxins and/or virulence factors was suggested [35, 65]. Indeed, the importance of alpha toxin in the pathogenesis of bovine necro-haemorrhagic enteritis was demonstrated in a calf intestinal loop model, by using different approaches. First, an alpha toxin-mutant strain was attenuated in its lesion-inducing potential in the intestinal loop model, whereas complementation of alpha toxin restored its ability to cause necro-haemorrhagic lesions [66, 67]. Next, when antisera containing antibodies against native alpha toxin were co-injected with *C. perfringens* in bovine intestinal loops, the lesion-inducing potential of *C.* *perfringens* was reduced [66]. Furthermore, when pure alpha toxin was injected in bovine intestinal loops, it caused epithelial cell detachment, villus tip blunting, erosion, mild inflammation and haemorrhages of the lamina propria, all events that are seen in natural cases of necro-haemorrhagic enteritis [68].

In addition to a confirmed involvement of alpha toxin in bovine necro-haemorrhagic enteritis, this toxin might also play a role in enteric diseases in other animals, including humans [64, 69–75]. When combining the research data on alpha toxin in enteric diseases conducted in different animal species, a pathological mechanism of alpha toxin in *C. perfringens* type A-associated enteric disorders can be proposed. Histopathologically all these intestinal disorders are characterized by damage to the tips of the villi or epithelial cell detachment, congestion of the capillaries, mucosal oedema and necrosis. In most cases, also haemorrhages and mucosal inflammation with concomitant influx of inflammatory cells is reported [1, 76–79]. For some of these pathological findings, there is indirect evidence that alpha toxin is responsible. In small intestine explants of rabbits incubated with alpha toxin, this toxin causes detachment of the epithelial cells at the tip of the villi [80]. Epithelial sloughing was also observed when alpha toxin was inoculated in bovine intestinal loops [68]. Alpha toxin is able to upregulate the matrix metalloproteinase (MMP) expression of the host as seen in vitro. This increased host MMP activity may be related to derangement of normal epithelial growth and increased degradation of subepithelial matrix, possibly explaining the observed epithelial detachment [81]. Additionally, alpha toxin induces the production of tumor necrosis factor-alpha (TNF-α) by mononuclear cells, which may contribute to epithelial sloughing. It has been shown that intraperitoneal or systemic administration of TNF-α to mice or intraduodenal TNF-α injection in rats induces pathological intestinal cell shedding and that dysregulated TNF-α production is highly important in driving epithelial damage as shown in mice [82, 83]. Another characteristic of *C. perfringens* type A-associated intestinal diseases that can be a result of the alpha toxin activity is the influx of inflammatory cells. Neutrophilic inflammation of the small intestine has been observed after intragastric administration of alpha toxin to neonatal piglets and after alpha toxin injection in small intestinal loops of rats, sheep and calves [68, 84, 85]. This trafficking of inflammatory cells to the infected tissues seems contradictory to the observations in gas gangrene, where the leukocytes are trapped inside the blood vessels. However, this difference may be related to the concentration of alpha toxin in the tissue. In gas gangrene, alpha toxin is produced in the tissue, leading to high toxin concentrations at the site of infection. This is in contrast to intestinal infections, where alpha toxin is produced by *C. perfringens* in the intestinal lumen and enters the mucosa through a currently unknown mechanism. Little is known about the permeability of the intestinal mucosa to alpha toxin, but it is likely that lower concentrations will be present in the intestinal mucosa than in the infected muscle tissue during gas gangrene [2]. Alpha toxin is well known to cause upregulation of adhesion molecules and IL-8 expression in endothelial cells and leukocytes [86, 87]. When present in excessively high concentrations as observed in mouse models of gas gangrene, these intercellular mediators alter the processes of leukocyte adherence and extravasation, resulting in impaired movement of inflammatory cells to the infected tissue [88]. However, physiological levels of upregulation lead to trafficking of neutrophils into the tissue space, as observed in intestinal *C. perfringens* type A infections. Furthermore, Otamiri et al. have shown that this neutrophil influx was caused by alpha toxin-induced activation of endogenous phospholipase A_2_ in the rat intestinal mucosa [85]. Activation of endogenous phospholipase A_2_ can result in the generation of highly pro-inflammatory lysophosphatidic acid which can damage the mucosa. The mucosal damage can be aggravated by oxidants, proteolytic enzymes and cytotoxic proteins originating from the infiltrated neutrophils, and may be associated with increased mucosal permeability. Indeed, an alpha toxin-triggered increase in intestinal permeability was shown in rats, rabbits, sheep and chickens [75, 80, 84, 85]. Additionally, alpha toxin induces the production of platelet-activating factor (PAF) and TNF–α by endothelial and mononuclear cells respectively, which likely contribute to the increased vascular permeability and oedema during *C. perfringens* infections [89, 90]. This increased vascular permeability may explain the haemorrhages observed after *C. perfringens* type A infections in some species. Indeed, haemorrhages of the lamina propria were reproduced after alpha toxin inoculation in ligated loops of the bovine small intestine [68]. Furthermore, the lethal effect of intra venous administration of alpha toxin to mice is closely related to the release of TNF-α from mononuclear cells into the bloodstream [91]. It should be noted that, although alpha toxin is described as a necrotizing toxin, no intestinal necrosis could be observed in any of the experimental models mentioned before. However, a mutant strain devoid of alpha toxin is attenuated in its lesion-inducing potential when injected in bovine intestinal loops [66, 67]. Together, these findings strongly suggest that alpha toxin is essential, but a synergism with other factors is needed to cause the fulminant necrosis seen in natural cases of necro-haemorrhagic enteritis [66, 68]. Up till now, the nature of the additional virulence factors is unknown, but there is some indirect evidence pointing towards certain toxins and enzymes.

### Perfringolysin O

Perfringolysin O is a cholesterol-dependent cytolysin which is produced by nearly all *C. perfringens* strains [92, 93]. This toxin is not considered essential for disease development, but seems to have an important accessory role in some diseases. Indeed, in a mouse model for gas gangrene it was found that perfringolysin O affects the host inflammatory response and is, at least partially, involved in tissue destruction. However, these effects were less pronounced than those elicited by alpha toxin and a synergistic effect between both toxins has been shown [89, 94, 95]. The role of perfringolysin O in *C. perfringens* type A-associated intestinal diseases in cattle is not yet fully understood. In a recent study antisera from calves immunized with a mixture of *C. perfringens* toxins were able to protect against intestinal necrosis when co-injected with *C. perfringens* in bovine intestinal loops [96]. Antibodies towards alpha toxin and perfringolysin O were identified as the most abundant antibodies in the protective immune sera, suggesting a possible role of both toxins in intestinal lesion development [96]. Verherstraeten et al. recently demonstrated a synergistic cytotoxic effect between alpha toxin and perfringolysin O to bovine endothelial cells. However, a perfringolysin O-deficient strain was still able to cause necrosis in calf ligated intestinal loops [67]. It should be noted that this does not exclude a possible accessory role of perfringolysin O in intestinal disease. Based on the knowledge obtained from gas gangrene, the effect of perfringolysin O is expected to be more subtle and further research should be focused on the host inflammatory response and more specific the neutrophil and macrophage influx into the lesions.

### A role for proteolytic or carbohydrate-active enzymes?

In addition to alpha toxin and perfringolysin O it cannot be excluded that the protection afforded by antisera derived from calves vaccinated with a mixture of *C. perfringens* toxins in bovine intestinal loops is due to other immunogenic proteins [96]. These additional virulence factors do not necessarily have to be toxins, but can equally well be proteins that confer a specific advantage to *C. perfringens* during intestinal colonization and/or infection.

#### Sialidases

*C. perfringens* needs to breach through the protective intestinal mucus layer before alpha toxin, either or not in synergy with other toxins, can act on the bovine intestinal tissue. The large, complex structure of mucins makes them targets for many proteases, glycosidases and sulphatases. Enzymatic digestion of the mucus layer provides access to readily available sources of carbon and enables bacteria to reach the epithelial surface. *C. perfringens* can produce many glycosidases which have a range of catalytic specificities that reflect their capacity to breakdown a diversity of host glycans [97–100]. Many of the studies concerning mucin-degrading enzymes were carried out specifically on sialidases. These enzymes cleave terminal sialic acids from sugar chains of glycoproteins, glycolipids, oligosaccharides, gangliosides and other sialoglycoconjugates. Sialic acids are especially abundant in the intestinal tract, where they are major constituents of mucins. In *C. perfringens* three sialidase enzymes have been reported, the large exo-sialidases NanI and NanJ, and a smaller intracellular NanH enzyme. Genome sequencing showed that the majority of strains carry all three sialidase-encoding genes [101]. The sialidases release sialic acid from higher order gangliosides and glycoproteins, probably for subsequent transport into the bacterial cell [102]. Furthermore, the release of sialic acid is an initial step in the sequential degradation of mucins, since the terminal location of sialic acid residues in the mucin oligosaccharide chains may prevent the action of other glycosidases [103]. In contrast with gut commensals, which appear to use sialidases primarily for nutrient acquisition, some pathogens, such as *C. perfringens*, also use sialidases to decrypt adhesins or toxin-binding sites [103]. Indeed, in studies on gas gangrene a synergy between alpha toxin and the NanI sialidase was observed [104, 105]. In these experiments alpha toxin had greater pathological effects on cultured cells that had been pretreated with NanI. Intramuscular injection of both alpha toxin and NanI in mice confirmed this synergy in vivo [104]. This enhancement of alpha toxin activity by NanI is dependent on the presence of gangliosides on the surface of the cell. Cleavage of sialic acid from these gangliosides, which protrude from the cell surface, most likely allows better interaction of alpha toxin with its substrates at the cellular surface [104, 105]. However, the use of either a *nan* *I*-mutant or *nan* *J*-mutant strain showed that large sialidases are not essential for virulence in a mouse model for gas gangrene [105]. This, however, does not exclude the possibility that sialidases are involved in the pathogenesis of gas gangrene, because subtle effects that might be mediated by the sialidases are masked in this model [49, 105]. In addition to a possible role in gas gangrene, the large sialidases may also be of importance during intestinal infections. Recent research suggests that NanI sialidase may contribute to intestinal attachment and colonization. This conclusion was based on the observation that NanI sialidase promotes the adherence of a *C.* *perfringens* type A, type C and type D strains to enterocyte host cells in vitro [106, 107]. Furthermore, pre-treatment of sensitive cells with NanI sialidase enhanced the subsequent binding and cytotoxic effects of epsilon toxin, suggesting that the large sialidases of *C.* *perfringens* can act in synergy with this toxin during type D enterotoxaemia [106]. However, no difference in in vitro mucin-degrading activities between *C. perfringens* strains originating either from bovine necro-haemorrhagic enteritis cases, from healthy cattle isolates or from other animal species could be demonstrated [108]. This, however, does not exclude a possible role of mucin-degrading enzymes in intestinal pathogenesis as the production of virulence factors in vitro does not necessarily reflect the in vivo situation, where contact with host tissue might alter the *C. perfringens* toxin production. Next, not only enzymatic differences but also functional differences such as enhanced substrate binding or reduced sensitivity to intestinal proteolytic inactivation might confer selective advantage to the producing strain. The exact role of sialidases and other mucin-degrading enzymes in *C. perfringens* type A-associated intestinal diseases has not been explored. Because it seems very likely that colonization and degradation of the small intestinal mucus layer are a prerequisite for the onset of necro-haemorrhagic enteritis, the glycosidases employed by *C. perfringens* to cope with the mucosal surface are probably of great importance and future research should focus on upregulation of these enzymes after contact with host tissue, the role of mucin-degrading enzymes in intestinal colonization and enzyme stability in the intestinal tract.

#### Hyaluronidases

Hyaluronan can form highly viscous solutions and is a major constituent of the extracellular matrix, especially in soft connective tissue [109]. The viscous consistency usually provides resistance to penetration of infectious agents and their extracellular products. Hyaluronidases are produced by a number of bacteria that cause infections at mucosal surfaces [110]. Hyaluronidase-mediated degradation of hyaluronan decreases the viscosity, which results in increased permeability of the connective tissues and potentially increased spread of microorganisms and toxins through the connective tissues [110, 111]. Alternatively, hyaluronidase may degrade hyaluronan cell coatings, thereby allowing direct contact between the pathogens and the host cell surfaces. Furthermore, the end products of hyaluronidase degradation are disaccharides which can be used as nutrients by the pathogen [110]. In *C.* *perfringens* 5 hyaluronidase genes are described (*nagH, nagI, nagJ, nagK* and *nagL*), which encode secreted enzymes [33]. Not much research has been done on the *C. perfringens* hyaluronidases. The best studied enzyme is mu toxin or NagH [112, 113]. By itself, mu toxin is a non-lethal toxin but it is thought to contribute to the pathogenesis of *C.* *perfringens* infections through the degradation of mucins and connective tissue [114]. Furthermore it facilitates the spread of alpha toxin, thereby potentiating its activity [112]. Because the *C.* *perfringens* hyaluronidases are not as well studied as the other *C.* *perfringens* toxins, no experimental evidence exists about the actual role of these enzymes in either gas gangrene or intestinal infections. Future research should elucidate whether contact with host cells causes upregulated hyaluronidase expression, and if bovine disease isolates show enhanced hyaluronidase activity or resistance to intestinal degradation as compared to other *C. perfringens* strains.

#### Collagenases

Collagen is widely distributed throughout the body and is an integral component of the connective tissues and the basal membranes. Collagen disruption by bacterial collagenases may result in the loss of tissue integrity and subsequent tissue necrosis [115]. *C.* *perfringens* can produce various collagenolytic enzymes with molecular masses ranging from ≈80 to ≈120 kDa [116, 117]. Historically, research was focused on the 80 kDa collagenase, which was designated as kappa toxin [116, 118, 119]. This 80 kDa collagenase was lethal for mice after intravenous injection. Furthermore, it has haemorrhagic and dermonecrotic activities and it can cause extensive connective tissue destruction, suggesting a potential role in the pathogenesis of gas gangrene [119]. However, a positive correlation was not always found between the virulence of *C.* *perfringens* and the ability to produce collagenase, and anti-collagenase was not effective in preventing experimental gas gangrene in guinea pigs, nor did it enhance the protective properties of anti-alpha toxin [120]. From 1994 onwards, research has switched from the 80 to the 120 kDa collagenase and the term kappa toxin is used to describe the 120 kDa protein [116, 121, 122]. It is suggested that the 80 kDa collagenase can be generated from the 120 kDa protein, but no experimental evidence exists to support this hypothesis [123, 124]. As described above, collagenases could play a role in clostridial virulence in terms of spreading toxins and bacterial cells to host tissue, and in tissue necrosis. The use of a *col* *A*-mutant *C.* *perfringens* strain revealed that its 120 kDa gene product is not essential for disease in a mouse model for gas gangrene [124]. However, studies using this model are limited given that the mouse gas gangrene model does not enable conclusions to be drawn about the early stages of the infection [49]. The role of *C. perfringens* collagenases in intestinal diseases is not yet explored. However, these enzymes are likely involved in multiple stages of necro-haemorrhagic enteritis: its action on the basal membrane might induce epithelial sloughing, whereas in later stage of disease, breakdown of the connective tissue might lead to massive tissue necrosis and the haemorrhagic nature of the 80 kDa enzyme might contribute to the observed haemorrhages.

## Pathogenesis: a hypothetical model

**Figure 3:**
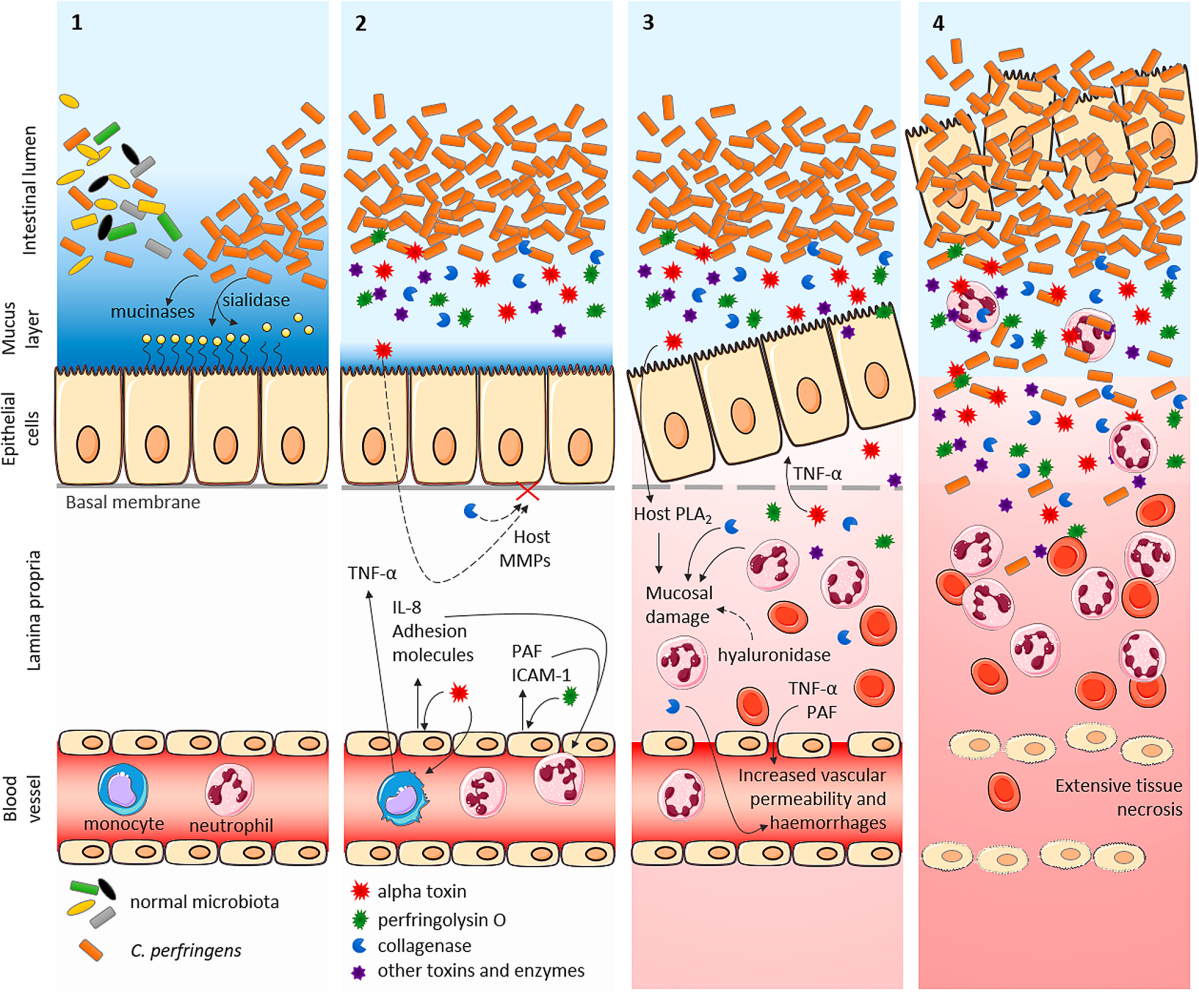
**Hypothesis on key events in** ***C. perfringens*** **type A-induced intestinal necrosis.** Key risk factors for the onset of disease are an intestinal environment that favours growth of *C. perfringens* and/or induces initial epithelial damage. **1** Disease starts with rapid, presumably mucus-associated, proliferation of *C. perfringens*. Production of a variety of mucin-degrading enzymes leads to breakdown of the protective mucus layer and sialidases remove sialic acid residues from the cells, making the epithelial cells easier to reach and unmasking potential binding sites for other *C. perfringens* toxins and enzymes. Furthermore, free sialic acid and mucin fragments provide a source of carbon and nitrogen, favouring further clostridial growth and toxin production. **2** *C. perfringens* produces a variety of toxins and enzymes. Alpha toxin and perfringolysin O stimulate endothelial cells for the production of IL-8, adhesion molecules (by alpha toxin) and ICAM-1 and PAF (by perfringolysin O), leading to trafficking of neutrophils into the tissue space. Furthermore alpha toxin induces TNF-α production by monocytes and may have an effect on host MMPs. **3** The earliest histopathological changes observed are epithelial sloughing and capillary congestion evolving to haemorrhages. Interestingly, the epithelial lining appears intact at this stage. Alpha toxin induces epithelial sloughing, probably through TNF-α and through host MMP activity on the basal membrane. Furthermore TNF-α and PAF likely contribute to the increased vascular permeability. *C. perfringens* collagenase has haemorrhagic activities and may be involved in the breakdown of the basal membrane and further connective tissue destruction. This mucosal damage is a result from various factors, including alpha toxin-induced activation of endogenous PLA_2_ and neutrophil-derived oxidants, proteolytic enzymes and cytotoxic proteins. **4** All these events eventually lead to fulminant intestinal necrosis and allow absorption of inflammatory cytokines (such as TNF-α) and toxins from the intestinal lumen into the systemic circulation, leading to shock and rapid death. Dashed arrow: hypothetical activities. Full arrow: proven intestinal activities.

A hypothesis on the key events in *C. perfringens* type A-induced intestinal necrosis is summarized in Figure 3. Bovine necro-haemorrhagic enteritis is a disease in which a concerted action of *C. perfringens* enzymes and toxins induces epithelial and vascular permeability. Subsequently the induced host responses and the action of bacterial components in the mucosa trigger intestinal inflammation, necrosis and haemorrhages. As *C. perfringens* type A strains are ubiquitous in the environment and are members of the normal intestinal microbiota, it is generally accepted that predisposing factors are required for *C. perfringens* to cause disease. Amongst the predisposing factors a high protein diet and stressful conditions are of major importance, but the exact mechanism behind this predisposition is still unclear. The most common hypothesis for the onset of *C. perfringens*-associated enteric diseases is the development of an intestinal environment that favours the growth of *C. perfringens* and triggers toxin production. However, when samples are taken shortly after death, no differences in intestinal *C. perfringens* counts were observed in calves who died from necro-haemorrhagic enteritis as compared to control calves [9, 27]. There is increasing evidence that *C. perfringens* toxin production is upregulated upon sensing of host tissue, a process that is probably regulated by quorum sensing mechanisms [125–127]. Together, this suggests that elevated numbers of *C. perfringens* in the intestinal lumen are no prerequisite for disease development and that overgrowth in proximity to the intestinal epithelium might be of greater importance. Certain feed components can alter intestinal mucus secretion and increase the intestinal mucosal permeability [37, 128, 129]. Furthermore, the predisposing effect of stressful conditions might be attributed to an increased intestinal permeability, and an impaired intestinal peristalsis which cancels the beneficial flushing effect of intestinal transit and therefore may contribute to bacterial overgrowth and mucus colonization [12, 39, 40, 42]. The increased mucosal permeability may facilitate toxin penetration into the intestinal mucosa, which is needed to reach their target cells. *C. perfringens* itself also produces various mucin-degrading enzymes, which may lead to breakdown of the protective mucus layer and concomitant disturbance of the gut barrier. *C. perfringens* sialidases might remove sialic acid residues from the cells, making the epithelial cells easier to reach and unmasking potential binding sites for other toxins and enzymes [103, 104, 106, 107]. Furthermore, the free sialic acid and mucin fragments provide an energy source for *C. perfringens* growth and further toxin production. The earliest histopathological changes are shedding of epithelial cells into the intestinal lumen and congestion of the capillaries. Interestingly, the epithelial cells are detached in seemingly intact strings, suggesting that the first pathologic events are located at the basal membrane. Alpha toxin induces epithelial sloughing, probably through TNF-α and through host MMP activity on the basal membrane. Indeed, elevated host collagenase activities were previously observed in intestinal tissue of broilers [45] and calves (unpublished data) challenged with *C. perfringens* type A strains. Additionally also *C. perfringens* collagenases may be involved in breakdown of the basal membrane. When the epithelial barrier is breached, the various *C. perfringens* toxins and enzymes can penetrate the mucosa and reach their respective targets. *C. perfringens* hyaluronidase activity might increase the mucosal permeability and facilitate the spread of toxins through the connective tissue [111, 114]. Alpha toxin stimulates endothelial cells for the production of the neutrophil chemoattractant, interleukin-8 (IL-8), and two vasoactive lipids, PAF and prostacyclin. Furthermore, alpha toxin also induces the upregulation of adhesion molecules, both in endothelial cells and neutrophils [86, 87]. Perfringolysin O enhances the expression of pro-adhesive molecules on leukocytes, as well as ICAM-1 (intracellular adhesion molecule 1) and PAF on endothelial cells [87, 130, 131]. Together, these effects elicited by both alpha toxin and perfringolysin O contribute to the observed trafficking of neutrophils into the tissue space. Furthermore TNF-α and PAF likely contribute to the increased vascular permeability. *C. perfringens* collagenase has haemorrhagic activities and may also be involved in further destruction of the connective tissue. Thus mucosal damage results from the combined action of various factors, including the alpha toxin-induced activation of endogenous phospholipase A_2_ (PLA_2_), activity of *C. perfringens* hyaluronidase and other toxins and enzymes, and neutrophil-derived reactive oxygen species, proteolytic enzymes and cytotoxic proteins. All these events eventually lead to fulminant intestinal necrosis and allow the diffusion of inflammatory cytokines (such as TNF-α) and toxins (e.g. LPS from Gram negative bacteria) from the intestine into the systemic circulation, leading to shock and rapid death. A systemic effect of alpha toxin in bovine intestinal diseases was never demonstrated and would not be considered great because alpha toxin is rapidly metabolised and eliminated from the blood stream [132].

## Diagnosis

Despite the advances in the understanding of *C. perfringens* type A-associated enteric diseases, bovine necro-haemorrhagic enteritis remains a substantial diagnostic challenge. Overall, diagnosis of bovine necro-haemorrhagic enteritis cannot be confirmed by a specific test. The confirmation of a clinical suspicion of enterotoxaemia often remains difficult, and in atypical clinical presentations the diagnosis is often questionable. Therefore, multiple diagnostic techniques, including necropsy, histology of the intestine and bacteriology (including cultures for other causes of enteritis) should be combined and samples should be taken from the cadaver as soon as possible, ideally within 15 min, to optimize chances for a correct diagnosis. Diagnosis of *C. perfringens* type A enteric diseases is complicated by the fact that type A strains are found in the gastrointestinal tract of healthy animals. Therefore, isolation of *C. perfringens* type A or detection of its major toxin, alpha toxin, from faeces or gastrointestinal content has little if any diagnostic value. Furthermore, *C. perfringens* strains isolated from necro-haemorrhagic enteritis cases cannot be differentiated from other *C. perfringens* type A strains in vitro with regard to their alpha toxin, perfringolysin O or proteolytic activities [108]. Enumeration of *C. perfringens* in the content of affected intestinal segments was historically recommended to confirm a clinical suspicion of necro-haemorrhagic enteritis. This practice was based on the presumption that clostridial enteric diseases are caused by a massive multiplication of *C. perfringens* in the gut, which was strengthened by a study suggesting higher *C. perfringens* load in intestinal content from calves that died from necro-haemorrhagic enteritis [133]. However, this study was biased by a large age difference between the control samples and the necro-haemorrhagic enteritis samples. This age difference will unavoidably imply dietary differences, which are known to influence the microbiota composition, questioning whether the observed differences in *C. perfringens* enumeration are really indicative for necro-haemorrhagic enteritis. Furthermore, *C. perfringens* multiplies in high numbers in the intestine as part of the post-mortem putrefaction process. More recently, enumeration of *C. perfringens* in necro-haemorrhagic enteritis samples and appropriate control samples revealed no differences in intestinal *C. perfringens* counts, even when samples were taken within 3 h after death, suggesting that *C. perfringens* enumeration in the intestinal content is not a valid diagnostic tool [9].

## Vaccination

Clostridial diseases are often rapidly fatal. Therefore vaccination is usually the only achievable intervention. Most available clostridial vaccines are combination vaccines against several clostridial species, often including toxoids derived from multiple toxinotypes of *C. perfringens*. Amongst the *C.* *perfringens* toxinotypes, type C and type D toxoids are almost always included in clostridial vaccines, whereas the other *C.* *perfringens* toxinotype toxoids are not always all included. In addition, toxoids from several other clostridial species are usually present in the vaccines: *Clostridium chauvoei*, *Clostridium novyi*, *Clostridium sordellii*, *Clostridium septicum* and *Clostridium tetani*. A few vaccines also include other bacteria such as *Mannheimia haemolytica* or enterotoxigenic *Escherichia coli* [1]. These toxoid-vaccines are made from culture supernatants which are inactivated, mostly using formaldehyde, and are subsequently subjected to an ultrafiltration purification process, which removes the bacterial cells and concentrates the desired antigens.

Until the recent demonstration of the essential role of alpha toxin in necro-haemorrhagic enteritis, not much attention was paid to alpha toxin in enteric diseases and its subsequent importance in vaccine composition. However, it is well known that the protective antigenicity of alpha toxin is easily destroyed by formaldehyde inactivation [134–138], possibly explaining why the current clostridial vaccines are unable to protect against bovine necro-haemorrhagic enteritis [29, 96]. Additional indirect evidence pointing towards the importance of alpha toxin as a vaccine antigen is the observation that calves in veal production systems do not develop an active immunity towards alpha toxin, when maternal immunity declines. This absence of antibody production after decay of maternal antibodies might explain why calves in veal production systems are at higher risk to develop necro-haemorrhagic enteritis than calves raised for beef production, in which a fluent transition from passive maternal to active immunity is observed [32, 36]. The C-terminal domain of alpha toxin is a promising candidate for future vaccine development [66]. Indeed, the C-terminal fragment of alpha toxin is able to mount an immune response in calves and the resulting antisera show some protective properties against both *C. perfringens*-induced cytotoxicity and intestinal necrosis [66, 139].

Nevertheless, even if alpha toxin is indispensable to cause necro-haemorrhagic lesions, the presence of alpha toxin alone seems insufficient to cause the fulminant necrosis seen in natural cases [66, 68]. Furthermore, when comparing antisera from calves immunized with alpha toxin alone versus antisera from calves vaccinated against a mixture of native *C.* *perfringens* toxins, the latter had a stronger ability to protect against *C.* *perfringens*-induced necrosis when co-injected with *C.* *perfringens* in bovine intestinal loops [66, 96]. Therefore other virulence factors are likely involved as well in the pathogenesis and might be needed as vaccine components to provide full protection against bovine necro-haemorrhagic enteritis. Up till now the nature of the additional antigens which are needed to provide this protection is not clear. One possible candidate to include in future vaccines is perfringolysin O. Indeed, antibodies against both alpha toxin and perfringolysin O were detected in calf antisera that were able to protect against *C. perfringens* challenges in the intestinal loop model [96]. Furthermore, vaccination of calves with the non-toxic, perfringolysin O derivative PFO^L491D^ either or not in combination with the non-toxic, C-terminal domain of alpha toxin, resulted in antibodies that were able to protect against the activities of the respective toxins in vitro [140]. More research is needed to elucidate whether addition of perfringolysin O as a vaccine antigen can increase the protective potential of the alpha toxin antisera against *C. perfringens*-induced intestinal lesions. Furthermore, it should be explored whether antibodies against other *C. perfringens* toxins or enzymes can also provide additional protection.

## Conclusions

The identification of alpha toxin as a key virulence factor in bovine necro-haemorrhagic enteritis provides some important novel insights. First, it clearly shows that the current view on *C. perfringens*-associated enteric diseases, which focusses mainly on plasmid-borne, disease-specific toxins is too strict and that alpha toxin can be important in enteric diseases. It is likely that even in diseases where other disease-specific toxins (e.g. NetB) are necessary to cause disease, alpha toxin might still be important in the pathogenesis. Second, the finding that alpha toxin is essential for necro-haemorrhagic enteritis has some major implications for vaccination strategies. The conformational epitopes of alpha toxin are important to induce a protective immune response and these epitopes are easily destroyed by formaldehyde. This adds to the understanding why current clostridial vaccines, which are based on formaldehyde inactivated toxins, don’t seem to provide protection against *C. perfringens* type A-associated intestinal diseases in calves. In order to protect animals against *C. perfringens* type A-associated enteric disorders, novel vaccines are needed. Alpha toxin will probably be a key component in these vaccines and the non-toxic C-terminal domain of alpha toxin may be a good candidate for further vaccine development. In addition, the ideal vaccine formulation should also contain other, yet unidentified, factors needed to provide full protection. These factors may be accessory toxins or enzymes involved in lesion induction, or factors that make the strains more adapted to the host environment. Next to vaccine formulation, also vaccination strategy may be important. Maternal immunization can be useful to protect young animals against clostridial abomasitis. An important risk period for the development of necro-haemorrhagic enteritis is situated near the end of the fattening period. In these older calves, maternal antibodies have declined and active immunization might be needed. Another important question is whether systemic immunity is sufficient to protect against *C. perfringens* type A-associated enteric diseases. Since *C.* *perfringens* is an enteric pathogen and given the local activity of its toxins, we could speculate that mucosal IgA plays a more important role than serum IgG in the protection against bovine necro-haemorrhagic enteritis. In cattle, no reports are found describing the mucosal IgA expression in the intestine during *C.* *perfringens* infection. Also for other species, the literature concerning this topic is scant. In humans, a correlation between the serum levels of IgA to alpha toxin and the faecal *C.* *perfringens* counts has been documented, but the relevance of this observation to provide protection against disease is not yet clear [141]. In chickens, it has been shown that systemic antibodies are able to reach the mucosal surface under inflammatory or necrotic conditions [137]. Furthermore, experimental animal work on intestinal *C.* *difficile* infections has shown that protection can be mediated through simple exudation of serum antitoxin IgG across the inflamed intestinal epithelium [142]. These observations point towards a serum IgG response as major influencer of protective immunity, but more research in cattle is needed to support this hypothesis. The ideal situation probably combines both systemic IgG as well as mucosal IgA immunity. This has been achieved using *Bacillus subtilis* spores as vaccine delivery agent. This organism is able to colonise the gut without causing disease. Oral immunization of mice with *B.* *subtilis* spores displaying the C-terminal fragment of alpha toxin on the spore surface, resulted in increased serum IgG levels and secretory IgA detected in saliva, faeces or lung wash samples [143]. In addition to the use of *B.* *subtilis* spores, also the use of other intestinal organisms as antigen carriers can be explored, such as, amongst others, the use of *Eimeria* [144] or *Salmonella* [145]. Next, it should be investigated whether immunity against alpha toxin alone is sufficient when both systemic IgG as well as mucosal IgA immunity is obtained.

A major problem hindering vaccine development is the lack of an in vivo model. Despite multiple attempts by different research groups to reproduce bovine necro-haemorrhagic enteritis in vivo, the intestinal loop model remains the system closest to an intact animal that is able to reproduce the lesions consistently [8, 35, 68, 132]. Because of the lack of an in vivo model, most research groups mainly focus on the development of an immune response as a readout for vaccine development [139]. However, antibody titres measured by ELISA are not a guarantee for protection and functional studies are more likely to predict the protective capacity of the elicited antibodies [96]. Indeed, screening of antibodies against the *C. perfringens*-induced cytotoxicity to bovine endothelial cells in vitro seems to give largely similar results as the protective effect of these antibodies against *C. perfringens*-induced intestinal lesions in bovine intestinal ligated loops [66, 96]. As the susceptibility to different *C. perfringens* toxins varies between host species and tissues, results from one species cannot be extrapolated to other hosts [68, 132, 146]. While awaiting development of new vaccines, close monitoring and control of predisposing factors remains the best means to prevent necro-haemorrhagic enteritis.

## Abbreviations

NetB: necrotic enteritis B-like toxin
MMP: matrix metalloproteinase
TNF-α: tumor necrosis factor-alpha
PAF: platelet activating factor
PLA_2_: phospholipase A_2_
IL-8: interleukin 8
ICAM-1: intracellular adhesion molecule 1
LPS: lipopolysaccharide
